# Comparison of circulating metabolite concentrations in dogs and cats when allowed to freely choose macronutrient intake

**DOI:** 10.1242/bio.036228

**Published:** 2018-09-26

**Authors:** Jean A. Hall, Matthew I. Jackson, Jodi C. Vondran, Melissa A. Vanchina, Dennis E. Jewell

**Affiliations:** 1 Department of Biomedical Sciences, Dryden Hall 206, College of Veterinary Medicine, Oregon State University, Corvallis, Oregon 97331-4802, USA; 2 Pet Nutrition Center, Hill's Pet Nutrition, Inc, 1035 NE 43rd Street, Topeka, Kansas 66617-1587, USA

**Keywords:** Cats, Circulating metabolites, Dogs, Food intake, Macronutrient intake

## Abstract

Food intake changes circulating metabolite concentrations. Thus, a comparison of circulating metabolites between dogs and cats is necessarily confounded by the composition of foods offered. The objective of this study was to determine differences between dogs and cats when given the opportunity to choose their own macronutrient intake. Four experimental foods with similar palatability, but varying in macronutrient content were prepared for dogs, and four for cats. Foods were available to dogs (*n*=17) for food intake once a day and to cats (*n*=27) at all times. Food 1 was high protein; Food 2 was high fat; Food 3 was high carbohydrates and Food 4 was balanced for macronutrients. By choosing a combination of foods, each animal could individually set its own macronutrient intake. Plasma metabolomics were determined after pets had consumed their food intake of choice for 28 days. Cats had higher concentrations of the essential amino acids histidine, isoleucine, phenylalanine and valine, but lower concentrations of lysine, methionine and threonine compared with dogs. Overall, non-essential amino acids were higher in cats. Regarding lipids, cats had increased concentrations of highly polyunsaturated fatty acids (PUFA) after 28 days, although arachidonic acid (AA) was consistently higher in dogs. Regarding circulating microbial metabolites, there was more stability for dogs compared with cats (none changed over time in dogs versus 42% changed in cats; *P*<0.01). Concentrations of urea cycle intermediates, antioxidants and methylated compounds were also different between species. In conclusion, metabolite differences between dogs and cats reflected differences in species and food choices.

## Abstract (precis)

**Summary:** Dogs and cats offered foods with different macronutrient composition, but equivalent palatability, have different circulating metabolite concentrations that reflect differences in species and food choices.

## INTRODUCTION

The taste preferences and nutritional requirements of cats and dogs are different. It is often stated that ‘cats are not small dogs’ and this colloquialism extends to the metabolic handling of macronutrient classes in general, as well as to specific micronutrient concentrations. It is well known that cats require a higher percentage of protein than dogs, with specific requirements for additional taurine. Cats have a low rate of taurine synthesis and large amounts are excreted into the bile because cats conjugate bile acids only with taurine, whereas dogs conjugate bile acids with both glycine and taurine (Armstrong et al., 2010). Cats consuming foods deficient in taurine may have impaired vision and can develop severe heart disease. Cats also have differences compared with dogs in their capacity to synthesize long chain essential fatty acids (FA) from shorter, more saturated lipid precursors present in dietary plant foodstuffs. For example, cats make limited amounts of arachidonic acid [AA; 20:4 (n-6)] from linoleic acid [LA; 18:2 (n-6)] presumably because they lack the Δ-6-desaturase enzyme (Sinclair et al., 1979), whereas dogs readily convert LA to AA. These, and other metabolic incongruities, form the basis for different essential FA requirements. Regarding carbohydrates, domesticated cats lack salivary amylase and have low activities of intestinal and pancreatic amylase (Kienzle, 1993a,b), whereas dogs have evolved an expanded genetic capacity for starch digestion concurrent with domestication. With respect to micronutrients, cats require a different form of vitamin A than do dogs. Dogs can use beta-carotene as a precursor for vitamin A synthesis, but cats cannot convert beta-carotene effectively, even though it is absorbed across the intestinal mucosa. Consequently, cats need retinol added to their food (Chew et al., 2000; Gershoff et al., 1957). Thus, cats have different amino acid, lipid, and carbohydrate, as well as vitamin requirements compared with dogs.

When dogs and cats are offered complete and balanced foods formulated with varying concentrations of protein, fat and carbohydrate, without controlling palatability, cats will choose a high protein food and dogs will choose a high fat food (Bradshaw, 2006; Hewson-Hughes et al., 2016, 2013, 2011). We previously reported (Hall et al., 2018) in the first part of this study that if foods are balanced for palatability and pets are given the opportunity to choose their preferred macronutrient intake based on physiologic needs, dogs on average consume most of their calories from fat (41%) and then carbohydrate (36%), whereas cats on average chose to consume most of their calories from carbohydrate (43%) and then protein (30%). Age and lean or fat body mass also influenced protein intake (Hall et al., 2018). The purpose of the second part of the study was to describe changes in metabolite concentrations when their preferred macronutrient intake was consumed.

Others have assessed the effects of dietary macronutrient intake on the plasma metabolome of healthy adult cats by feeding control, high-fat, high-protein or high-carbohydrate foods (Deng et al., 2014), and shown that dietary changes primarily affect markers of amino acid and lipid metabolism, but may also alter concentrations of metabolites produced by gut microbial metabolism. Nutritional metabolomics studies in cats and dogs have been reviewed (Allaway, 2015) and show that in addition to the proportion of macronutrients consumed, the plasma metabolome is also dependent on the individual (Colyer et al., 2011), breed (Lloyd et al., 2017), gender and age (Allaway et al., 2016), as well as caloric intake (Wang et al., 2007) and dietary supplementation, for example with glucose supplementation (Allaway et al., 2013). In the design of many feeding studies, a comparison of the circulating metabolomes of dogs and cats is necessarily confounded by offering different foods to each species. Although, feeding both species the same food may be experimentally correct (Allaway et al., 2013; Colyer et al., 2011), the metabolome data is biologically compromised and of little value for comparative physiology because it forces one species to consume a food that it would not normally consume if allowed to choose a food on the basis of physiological needs. Clearly, if both species are fed the same food, one species' metabolic processes will be subjected to aberrant nutritional constraints or burdens. By contrast, the objective of this study was to determine the differences in circulating metabolite concentrations of dogs and cats when given the opportunity to choose their own macronutrient intake. Our goal was to identify differences in circulating metabolite concentrations between dogs and cats after a 28-day period of free-choice macronutrient intake. Dogs and cats were allowed to consume a self-selected mixture from one of four foods of varying macronutrient makeup that were balanced for palatability. Our colleagues (Colyer et al., 2011) have shown in both dogs and cats that concentrations of metabolites in fasting plasma samples are reproducible over three collection days, several days apart, and representative of the individual, and that a 2-week feeding period is a suitable adaptation period to provide a consistent fasted plasma metabolome profile. We hypothesized that the self-selected macronutrient preferences noted in our first study (Hall et al., 2018) would manifest as observable changes in circulating metabolomic products.

## RESULTS AND DISCUSSION

Canine and feline metabolite concentrations were compared to baseline values, respectively, after pets were given the opportunity to choose their own macronutrient intake for 28 days. At baseline, pets were enrolled into the study based on availability and therefore consisted of a random selection of pets routinely assigned to palatability trials. Thus, these pets had been consuming many types of commercial and non-commercial foods of varying nutrient compositions, including dry and canned foods. All these foods met the requirements established by the Association of American Feed Control Officials (AAFCO) for complete and balanced pet foods for adult dogs and cats. We deemed it conceptually challenging and of dubious biological merit to select a single baseline food for comparison to the food mixture chosen by each pet when given the opportunity to select their own macronutrient intake. Rather, we tested the food mixture chosen by each pet after the 28-day feeding period against a sum composite of foods typical pets may be fed, given that all baseline foods were complete and balanced foods. Metabolite concentrations that were significantly altered as a result of food intake over a 28-day feeding period are highlighted below for amino acids, lipids and carbohydrates. In addition, significantly altered concentrations of urea cycle intermediates, antioxidants, methylated compounds and microbial metabolites are summarized.

In total, 571 metabolites were detected across all species and test times. Of these, 262 circulating metabolites changed in cats when offered the opportunity to select a diet from foods with varying macronutrients (at *P*<0.05). Amongst these feline metabolites, 145 increased and 117 decreased. In contrast, in dogs only 48 metabolites met the same statistical criteria, with more than double the number of metabolites increasing (34) versus decreasing (14). There were 435 metabolites whose concentrations were different between dogs and cats at baseline, and of these, 199 were increased in dogs compared with cats, where 236 were decreased. After the free-choice feeding period, 441 metabolites differed between dogs and cats, with 201 increased in dogs compared with cats and 240 decreased. As assessed by Mixed Model, there were 453 metabolites exhibiting a species effect, 203 metabolites manifesting a diet choice/time effect and 194 metabolites having an interaction between species type and diet/time.

**Fig. 1.**
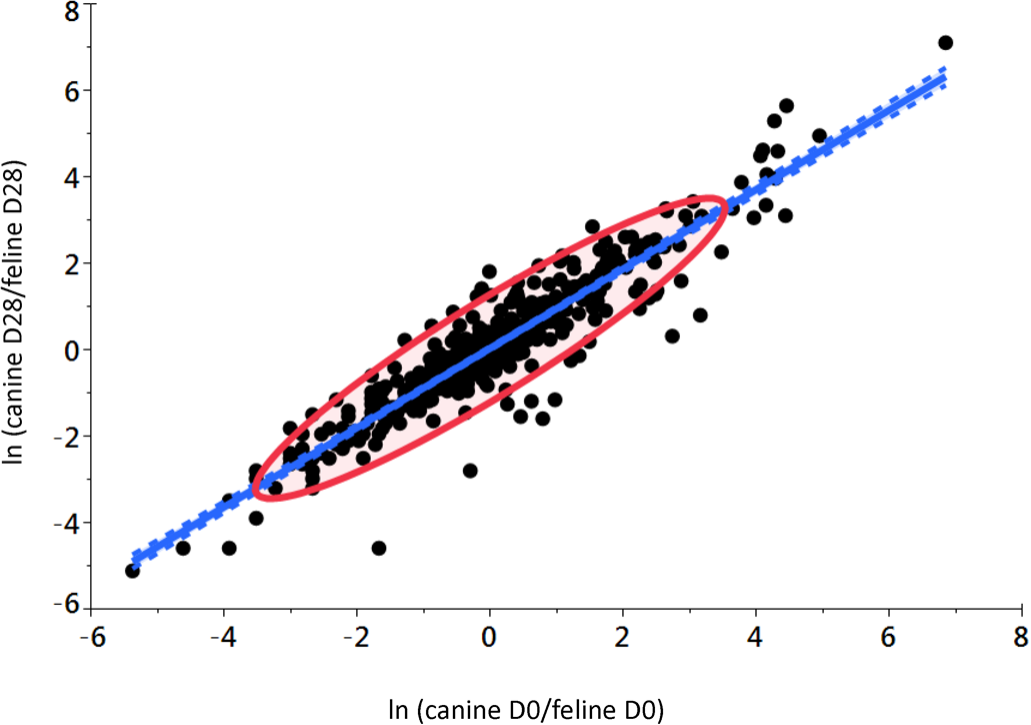
**Correlation of ratios of canine to feline metabolite concentrations at the end of the 28-day free-choice feeding period (D28) with baseline ratios of canine to feline metabolite concentrations (D0).** In general, dog to cat species ratios for individual metabolites after pets chose their preferred macronutrient intake for 28 days were highly correlated with dog and cat ratios for those same metabolites at baseline (571 metabolites; 17 dogs; 27 cats; *R^2^*=0.87; slope=0.92; *P*_Linear_ <0.001). The blue shaded area associated with the dotted lines along the length of this correlation line is the 95% confidence interval of the line of best fit. The elliptical red circle surrounds the 95% probability area of the data points themselves.

The strength of the species effect was further explored by comparing ratios of dog metabolite concentrations to cat metabolite concentrations at the end of the 28-day free-choice feeding period and examining whether these dog to cat species ratios at the end of the 28-day free-choice feeding period had changed significantly from baseline ratios of dog to cat metabolite concentrations. In general, dog to cat ratios for individual metabolites after pets chose their preferred macronutrient intake for 28 days were highly correlated dog to cat ratios for those same metabolites at baseline (571 metabolites; 17 dogs; 27 cats; *R*^2^=0.87; slope=0.92; *P*_Linear_ <0.001; Fig. 1). Intriguing off-linear metabolites in each of the macronutrient classes are discussed below.

Summing up, there were changes in circulating metabolite concentrations after 28 days in dogs and cats that were given the opportunity to choose their own macronutrient intake, but cats presented nearly seven times more changed metabolites after the free-choice food intake period compared with dogs. There were also species differences for dogs and cats in many circulating metabolite concentrations. Usually the ratio of dog to cat metabolite concentrations on day 0 were similar to those at day 28, but in some cases food choices altered the ratio at day 28 compared with the baseline ratio. In many cases, species differences after the free-choice period were driven by a change in cats rather than dogs. Interesting changes are discussed below according to macronutrient classes.

### Amino acids

Diet associated plasma metabolite profiles for essential, conditionally essential and nonessential amino acids for dogs and cats at baseline and after a 28-day feeding period, when given the opportunity to choose their own macronutrient intake, are shown in Table 1. For dogs, there were no significant changes in essential amino acid, conditionally essential amino acid or nonessential amino acid concentrations over the 28-day period compared with baseline. In cats, essential amino acid concentrations decreased after the 28-day free-choice feeding period for histidine, methionine, threonine, tryptophan and valine (compared with baseline concentrations. Conditionally essential amino acid concentrations decreased over the 28-day period for cystine (more stable, oxidized amino acid formed from two cysteine molecules linked by a disulfide bond), glutamine and tyrosine; taurine increased. Nonessential amino acid concentrations decreased over the 28-day period for alanine, asparagine and proline, but increased for glutamate.

**Table 1.**
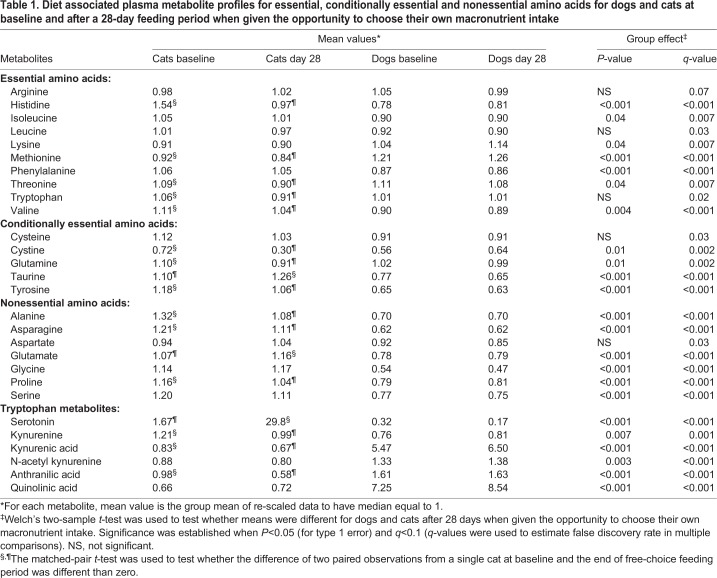
**Diet associated plasma metabolite profiles for essential, conditionally essential and nonessential amino acids for dogs and cats at baseline and after a 28-day feeding period when given the opportunity to choose their own macronutrient intake**

Comparing dogs with cats after consuming their preferred macronutrient intake for 28 days, dogs had lower concentrations for the essential amino acids histidine, isoleucine, phenylalanine and valine, for the conditionally essential amino acids tyrosine and taurine, and for the nonessential amino acids alanine, asparagine, glutamate, glycine, proline and serine. On the other hand, dogs had higher concentrations for the essential amino acids lysine, methionine and threonine and for the conditionally essential amino acids cystine and glutamine.

Overall, when dogs were allowed to choose the macronutrient composition of their food, concentrations of circulating amino acids did not significantly change from baseline. In cats, of the 12 amino acids whose concentrations changed significantly from baseline after free-choice food intake, only glutamate and taurine increased; all other amino acid concentrations decreased. Thus, it would appear that cats do not have an innate food selection ‘drive’ to maintain or increase circulating amino acid concentrations above the concentrations that manifest when consuming representative complete and balanced foods. Interestingly, although 3/10 essential (methionine, lysine and threonine) and 2/4 conditionally essential amino acids (cystine and glutamine) were higher in concentration in dogs than cats, cats manifested higher concentrations of 6/7 non-essential amino acids (alanine, asparagine, glutamate, glycine, proline and serine). The seventh, aspartate, was numerically but not significantly higher in cats. The fact that cats maintained consistently higher concentrations of circulating non-essential amino acids may reflect this species' propensity to utilize amino acids in place of carbohydrate for energy requirements (Macdonald et al., 1984b); each of these non-essential amino acids is glucogenic (Armstrong et al., 2010). Cats had higher concentrations compared with dogs of the essential amino acids histidine, isoleucine, phenylalanine and valine, and the conditionally essential amino acid tyrosine; these are also glucogenic amino acids. Although they are few in number, it is worth noting that neither of the two strictly ketogenic amino acids (leucine nor lysine) were higher in cats and, in fact, lysine concentrations were higher in dogs. Together, these metabolomics data strengthen our understanding that cats fuel their energy needs with a metabolic flow of glucose derived from non-essential amino acids.

Precipitated by the observation that circulating tryptophan concentrations decreased in cats after the 28-day free choice feeding period, metabolite partitioning into the serotonin and kynurenine branches of tryptophan metabolism was assessed. These pathways represent behavioral and immunologic outcomes of tryptophan metabolism (Gao et al., 2018; Gibson, 2018). Based on the magnitude of response, serotonin was the most increased circulating metabolite after the free choice feeding period (Fig. 1, this is evident as the most off-slope metabolite). This neuromodulator was not significantly altered in dogs after the free choice feeding period, consistent with tryptophan concentrations also being unchanged in this species. Serotonin production in cats is associated with a lag in manifestation of defensive rage behavior upon exposure to inflammatory microbial metabolites (Bhatt et al., 2009), and interventions using diets modified in tryptophan and large neutral amino acids concentrations has been attempted to alter serotonin metabolism and produce desirable behavioral changes (Miyaji et al., 2015). In this light, it is intriguing that cats selected foods containing a composition of amino acids that increased their concentrations of serotonin. Immunologic disease in cats is also associated with altered tryptophan metabolism and increased concentrations of kynurenine, with enhanced catabolism of tryptophan through this pathway proposed (Kenny et al., 2007). Cats manifested significantly decreased concentrations of three out of five observed metabolites of the kynurenine pathway after the free choice feeding period. Early pathway metabolites kynurenine and kynurenic acid, but not N-acetyl kynurenine, were significantly decreased. Regarding the two late pathway metabolites, anthranilic acid, but not quinolinic acid, was significantly decreased. None of these kynurenine pathway metabolites were significantly changed in dogs after the free choice feeding period. Taken together, it would appear that cats selected food compositions that predisposed them towards increased tryptophan catabolism, with increased serotonin concentrations occurring at the expense of kynurenine.

Taurine concentrations were increased in cats as a result of free-choice food intake likely because concentrations of taurine were higher in all food choices in this study compared with pre-trial foods. Taurine is a beta-amino acid that contains a sulfonic acid group, although it is not an amino acid in the usual biochemical meaning of the term, the latter referring to compounds containing both an amino and a carboxyl group. Taurine is essential in foods for cats because the feline liver has a limited capacity to synthesize taurine, and cats have an obligate loss of taurine in the enterohepatic circulation of bile acids because of microbial degradation in the intestinal tract (Armstrong et al., 2010). Taurine is conditionally essential for dogs. Taurine is not incorporated into proteins nor degraded by mammalian tissues (Armstrong et al., 2010). It is abundant as a free amino acid in the natural food of cats, such as small rodents and birds (Armstrong et al., 2010). Our results suggest that cats have a disposition to maintain higher circulating concentrations of taurine when presented with foods containing higher taurine concentrations.

### Polyunsaturated (n-3) and (n-6) fatty acids

In dogs, none of the (n-3) and (n-6) PUFA changed significantly over the 28-day feeding period compared with baseline concentrations (Table 2). On the other hand, in cats there were major increases in (n-3) PUFA concentrations after the 28-day feeding period compared with baseline concentrations for α-linolenate [18:3 (n-3)] or γ-linolenate [18:3 (n-6)], which were not distinguished from one another, stearidonate [18:4 (n-3)], eicosapentaenoate [EPA; 20:5 (n-3)], (n-3) docosapentaenoate [DPA; 22:5 (n-3)] and docosahexaenoate [DHA; 22:6 (n-3)]; and in (n-6) PUFA concentrations for linoleate [18:2 (n-6)], dihomo-linoleate [20:2 (n-6)], arachidonate [AA; 20:4 (n-6)], adrenate [22:4 (n-6)] and (n-6) docosapentaenoate [DPA; 22:5 (n-6)].

**Table 2.**
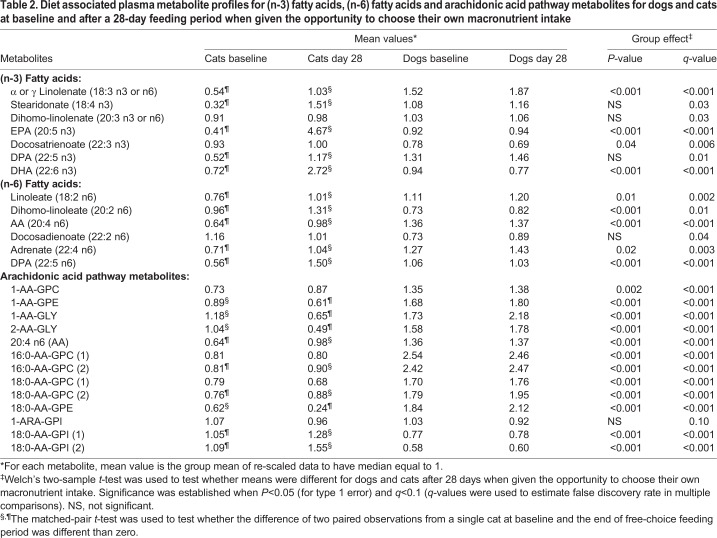
**Diet associated plasma metabolite profiles for (n-3) fatty acids, (n-6) fatty acids and arachidonic acid pathway metabolites for dogs and cats at baseline and after a 28-day feeding period when given the opportunity to choose their own macronutrient intake**

In cats but not dogs, multiple metabolite concentrations within the arachidonic acid pathway changed over the 28-day period compared with baseline concentrations. The following metabolites were decreased: 1-arachidonoyl-glycerophosphoethanolamine [1-AA-GPE], 1-arachidonoylglycerol [1-AA-GLY], 2-arachidonoylglycerol [2-AA-GLY] and stearoyl-arachidonoyl-glycerophosphoethanolamine [18:0-AA-GPE]. The following metabolite concentrations were increased: AA, palmitoyl-arachidonoyl-glycerophosphocholine (2) [16:0-AA-GPC (2)], stearoyl-arachidonoyl-glycerophosphocholine (2) [18:0-AA-GPC (2)], stearoyl-arachidonoyl-glycerophosphoinositol (1) [18:0-AA-GPI (1)] and stearoyl-arachidonoyl-glycerophosphoinositol (2) [18:0-AA-GPI (2)]. In general, arachidonoyl-containing glyceryl and ethanolamine complex lipids decreased in concentration while the equivalent choline and inositol congeners were increased.

Comparing dogs with cats for the (n-3) FA, after consuming their preferred macronutrient intake for 28 days dogs had lower concentrations for EPA, docosatrienoate [22:3 (n-3)] and DHA; and dogs had higher concentrations for α-linolenate [18:3 (n-3)] or γ-linolenate [18:3 (n-6], which were not distinguished from one another. Comparing dogs with cats for the (n-6) FA, after consuming their preferred macronutrient intake for 28 days dogs had lower concentrations for dihomo-linoleate [20:2 (n-6)] and DPA [22:5 (n-6)]; and dogs had increased linoleate, AA and adrenate.

Regarding metabolite concentrations within the arachidonic acid pathway after consuming their preferred macronutrient intake for 28 days, dogs had higher concentrations of 1-arachidonoyl-glycerophosphocholine [1-AA-GPC], 1-AA-GPE, 1-AA-GLY, 2-AA-GLY, AA, palmitoyl-arachidonoyl-glycerophosphocholine (1) [16:0-AA-GPC (1)], 16:0-AA-GPC (2), 18:0-AA-GPC (1), 18:0-AA-GPC (2) and 18:0-AA-GPE. In contrast dogs had lower concentrations of the inositol-containing lipids 18:0-AA-GPI (1) and 18:0-AA-GPI (2).

Overall, when dogs and cats were allowed to choose the macronutrient composition of their food, the food choices that cats made resulted in higher concentrations of circulating linolenate and linoleate. The food choices dogs made did not impact circulating concentrations of these FA. The metabolic impact of the choices cats made also affected the long-chain FA with higher degrees of unsaturation. For example, the (n-3) FA, stearidonate, which is the metabolic intermediate between linolenate and EPA was significantly increased during the free-choice period, as were concentrations of EPA, (n-3) DPA and DHA. Similarly, for the (n-6) series FA, increased linoleate concentrations resulted in increased dihomolinoleate. Although more ambiguous than the case for (n-3) FA elongation (because AA was present in foods consumed during the 28-day free-choice feeding period), AA and (n-6) DPA concentrations were increased consistent with chain elongation from linoleate and dihomolinoleate. Given the lack of significant sources of EPA and DHA in the foods offered to cats and dogs (foods did not have added fish oil; foods contained <0.01% EPA and DHA by formula calculation), these results demonstrate the cat's capacity to metabolize dietary linolenate to long-chain PUFA essential to health, i.e. the cat's ability to produce EPA and DHA from dietary precursors is robust. Whereas supplemental dietary EPA and DHA have been shown to have health benefits in cats (Hall et al., 2017, 2016, 2014), these data suggest that cats have a significant ability to synthesize them as well. This is in contrast to the observation that cats have a nutritional requirement for EPA and DHA because they have a limited ability to synthesize them (MacDonald et al., 1984a,b). Others have also shown that cats can produce higher order PUFA from linolenate, albeit less efficiently compared with dogs (Pawlosky et al., 1994).

The food choices that cats made decreased dog to cat species ratios for circulating AA, (n-6) DPA, EPA and (n-3) DHA that were present at baseline. Both (n-3) and (n-6) DPA were initially higher in dogs, but after the free-choice feeding period, cats manifested higher concentrations of these FA. Furthermore, EPA concentrations, which were initially higher in dogs became higher in cats following the free-choice feeding period. The same was true for DHA concentrations.

Arachidonate concentrations were consistently higher in dogs compared with cats at baseline and after 28 days of free-choice food consumption. This was especially evident in higher order lipid species incorporating AA. All measured glyceryl, GPC and GPE lipids containing AA were present at higher concentrations in dogs compared with cats. Counterintuitively, of the three measured AA-containing GPI metabolites, one was not significantly different between dog and cat species, and the other two had lower concentrations in dogs compared with cats. Accordingly, circulating myo-inositol concentrations (data not shown) were lower in dogs at baseline and after 28-days free-choice food intake (both *P*<0.001), as were GPI concentrations (data not shown) at baseline (*P*=0.02). The dog and cat species difference in apportioning AA into higher order lipids warrants further investigation. We have previously shown that dietary enrichment of dog foods with fish oil alters circulating metabolomic profiles of lysophospholipids, decreasing AA-containing GPE metabolites and increasing higher order DHA containing GPC metabolites (Hall et al., 2011). Additionally, others have shown that the individual is an important driver of variance in plasma metabolites, and that specific metabolites, primarily lipids, may be differentially regulated by individuals in dog and cat species (Colyer et al., 2011).

### Glycolysis and tricarboxylic acid (TCA or Krebs) cycle metabolites

Glucose concentrations did not vary for dogs or cats (Table 3) after consuming their preferred macronutrient intake for 28 days. Although lactate and pyruvate did not change after the 28-day period from baseline for dogs, lactate increased and pyruvate decreased over the 28-day period in cats. Dogs had lower concentrations of lactate after free-choice food consumption compared with cats.

**Table 3.**
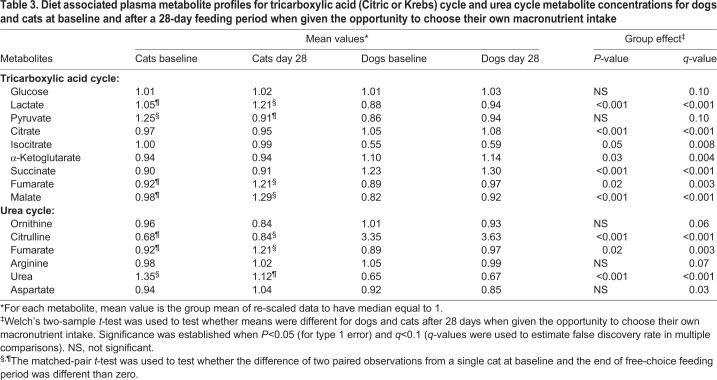
**Diet associated plasma metabolite profiles for tricarboxylic acid (Citric or Krebs) cycle and urea cycle metabolite concentrations for dogs and cats at baseline and after a 28-day feeding period when given the opportunity to choose their own macronutrient intake**

Regarding TCA cycle intermediates; in dogs, there were no changes over the 28-day period compared with baseline concentrations. In cats, after consuming their preferred macronutrient intake for 28 days, fumarate and malate concentrations increased compared with baseline concentrations. After consuming their preferred macronutrient intake for 28 days, dogs had lower concentrations of isocitrate, fumarate and malate, and higher concentrations of citrate, α-ketoglutarate and succinate.

It is important to note that dogs and cats maintained glucose homeostasis regardless of their preferred macronutrient intake over the 28-day free-choice feeding period. In dogs, lactate and pyruvate concentrations did not change over the 28-day period. However, in cats lactate concentrations increased and pyruvate concentrations decreased, perhaps indicating reliance on the Cori cycle to convert lactate produced by anaerobic glycolysis in muscles to glucose in the liver via intermediacy of pyruvate.

We have previously reported (Hall et al., 2018) that cats choose a broader range of protein intake compared with dogs, and variation in protein intake is offset primarily by carbohydrate intake, with minimal change in fat intake. The high protein requirement in cats may be the result of a high metabolic demand for glucose that must be met by amino acid-based gluconeogenesis (Eisert, 2011). Indeed, higher activities of rate-limiting enzymes of gluconeogenesis, i.e. pyruvate carboxylase, fructose-1,6-biphosphatase and glucose-6-phosphatase, have been demonstrated in feline liver compared with canine liver (Tanaka et al., 2005; Washizu et al., 1999).

Comparing dogs with cats after consuming their preferred macronutrient intake for 28 days, cats had higher concentrations of isocitrate, fumarate and malate concentrations. Isocitrate dehydrogenase catalyzes an oxidation reduction in which isocitrate is converted to α-ketoglutarate and carbon dioxide. It is the first of four oxidative steps and the key rate-limiting step of the TCA cycle. Fumarate and malate are two TCA cycle intermediates formed in the latter steps of the TCA cycle. The response to free-choice food intake from baseline to day 28 showed decreased ratios of fumarate and malate concentrations between dogs and cats, the result of higher concentrations of these TCA intermediates in cats.

### Urea cycle metabolites

Concentrations of urea cycle intermediates were evaluated for stability over the 28-day period when dogs and cats were allowed to consume their preferred macronutrient intake (Table 3). In dogs, there were no significant changes after consuming their preferred macronutrient intake for 28 days. In cats, citrulline and fumarate increased after consuming their preferred macronutrient intake for 28 days, whereas urea decreased. Compared with cats, at the end of the 28-day free-choice feeding period dogs had increased citrulline and decreased urea and fumarate concentrations.

In summary, for dogs there were no significant changes in urea cycle intermediates after consuming their preferred macronutrient intake for 28 days, whereas in cats urea concentrations decreased, and citrulline and fumarate concentrations increased after consuming their preferred macronutrient intake for 28 days. Decreasing urea concentrations in cats at 28 days implies that protein intake had declined as a result of free-choice feeding behavior. In cats, circulating citrulline concentrations are very low compared with other species (Levillain et al., 1996).

### Antioxidant metabolites

Concentrations of the tocopherols and their metabolites, and glutathione and its metabolites, were evaluated for each species when dogs and cats were allowed to consume their preferred macronutrient intake (Table 4). In dogs, the α-tocopherol metabolite α-CEHC [2,5,7,8-tetramethyl-2-(2'-carboxyethyl)-6-hydroxychroman] glucuronide decreased after consuming their preferred macronutrient intake for 28 days. In cats, alpha-tocopherol and the α-tocopherol metabolite α-CEHC-sulfate increased, whereas in the glutathione pathway cystine and the glutathione metabolites cystine-glutathione disulfide and 5-oxoproline decreased. In cats the glutathione biosynthesis pathway-related metabolites 2-hydroxybutyrate (AHB) and ophthalmate increased.

**Table 4.**
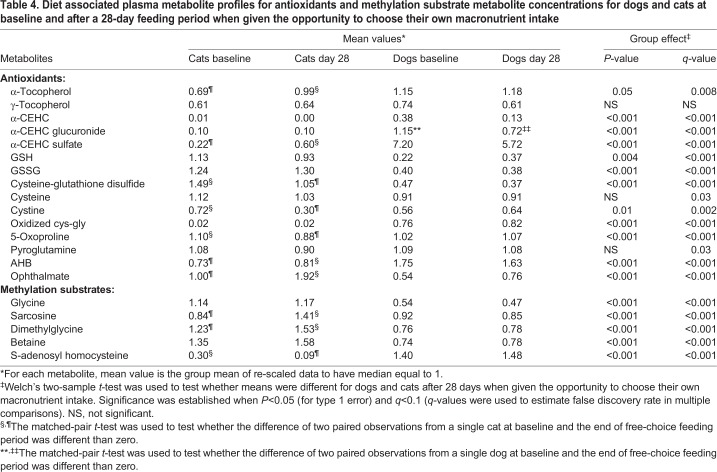
**Diet associated plasma metabolite profiles for antioxidants and methylation substrate metabolite concentrations for dogs and cats at baseline and after a 28-day feeding period when given the opportunity to choose their own macronutrient intake**

After consuming their preferred macronutrient intake for 28 days, dogs exhibited increased concentrations of alpha-tocopherol, alpha-CEHC, alpha-CEHC glucuronide and alpha-CEHC sulfate relative to cats. After consuming their preferred macronutrient intake for 28 days, dogs had decreased concentrations of reduced glutathione (GSH), oxidized glutathione (GSSG), cysteine-glutathione disulfide and ophthalmate. At the same time, dogs had increased concentrations of cystine, oxidized cys-gly, 5-oxoproline and AHB.

In summary, after dogs and cats were allowed to choose the macronutrient composition of their food for 28 days, dogs had higher concentrations of α-tocopherol and cats had higher concentrations of both oxidized and reduced glutathione. Yet, when the overall tocopherol and glutathione metabolic pathways were surveyed, dogs exhibited a higher oxidative burden than cats, both at baseline and after the free-choice feeding period. For example, the tocopherol-derived oxidized-chromane α-CEHC and its α-CEHC glucuronide and α-CEHC sulfate phase-2 detoxification end products, all were of higher concentration in dogs compared with cats by an order of magnitude.

Although it is difficult to assess quantitative reduction potentials of thiol-disulfide redox couples with this experimental design, total glutathione (both oxidized and reduced forms) was lower in concentration in dogs, whereas oxidized cys-gly was higher in concentration in dogs compared with cats. From the dog's perspective, higher concentrations of oxidized cys-gly and 5-oxoproline, the latter potentially derived from the enzymatic activity of gamma-glutamylcyclotransferase (EC 2.3.2.4), both have an inhibitory action on the cellular capacity to maintain appropriate GSH redox tone and export GSH into circulation (De Donatis et al., 2010). This may explain the decreased circulating glutathione concentrations observed in dogs relative to their feline counterparts.

Dogs also demonstrated higher concentrations of AHB, indicating that the transsulfuration pathway was more upregulated in dogs compared with cats. A caveat to this interpretation is that higher concentrations of AHB may reflect some contribution from threonine catabolism. There was, however, no statistical correlation between AHB, or its oxidized congener alpha ketobutyrate, and circulating threonine concentrations in dogs, lending credence to the interpretation that AHB is derived from the transsulfuration pathway. The transsulfuration pathway is a metabolic pathway involving the production of cysteine from homocysteine, through the intermediate cystathionine.

Ophthalmate is a tripeptide analog of GSH, with α-aminobutyrate in place of cysteine. It is produced in place of GSH when cysteine is limiting, but, lacking a thiol, it has no redox capacity. Ophthamate was increased in cats relative to dogs. Interestingly, this was paralleled by a strong preference in cats for foods that decreased circulating cysteine. Also, cats, relative to dogs, had higher total glutathione concentrations (e.g. GSH, GSSG and GSSC were all in higher concentrations in cats compared with dogs). Taken together, it appears that cats have a high demand for GSH, but when given a choice they decrease their intake of foods that would otherwise support GSH synthesis, in order to maintain circulating cyst(e)ine. The consequence of divorcing their metabolic propensity for GSH synthesis for maintenance of adequate cyst(e)ine availability is an increase in production of the metabolic dud ophthalmate.

### Glycine methylated metabolites

Concentrations of glycine, N-methylglycine (sarcosine), di-methylglycine and tri-methylglycine (betaine) were evaluated for stability over the 28-day period when dogs and cats were allowed to consume their preferred macronutrient intake (Table 4). In dogs, there were no significant changes after consuming their preferred macronutrient intake for 28 days. In cats, N-methylglycine and di-methylglycine increased after consuming their preferred macronutrient intake for 28 days.

Comparing dogs and cats after the 28-day free-choice feeding period, glycine, N-methylglycine, di-methylglycine and tri-methylglycine were all decreased. Concentrations of S-adenosylhomocysteine (SAH) were unchanged in dogs after consuming their preferred macronutrient intake for 28 days, whereas in cats concentrations decreased, consistent with nutritional enhancement of methylation capacity. Dogs had higher concentrations of SAH after the 28-day free-choice feeding period.

In summary, cats had consistently higher concentrations of components of methylation metabolism. Glycine, as well as its di-methyl and tri-methyl congeners, were found at higher concentrations in cats compared with dogs at baseline, and after free-choice feeding concentrations of glycine and mono-methylglycine were further increased in cats.

The first step in mammalian methionine metabolism, reviewed in (Verbrugghe and Bakovic, 2013) is conversion of methionine and ATP to *S*-adenosylmethionine, a universal methyl donor for all methylation reactions. The methyl group of *S*-adenosylmethionine is transferred to a variety of methyl acceptors, including nucleic acids, proteins, lipids and secondary metabolites, with formation of *S*-adenosylhomocysteine (SAH). Once the methyl group is transferred to a substrate by the appropriate methyltransferase, the SAH is rapidly hydrolysed to homocysteine and adenosine. Homocysteine may be re-methylated to regenerate methionine using folate, i.e. a methyl group is transferred from activated, methylated folate to homocysteine via the intermediary of methylcobalamin. An alternative, folate-independent pathway utilizes tri-methylglycine (betaine) derived from oxidation of choline, as a methyl donor to produce methionine and dimethylglycine. Furthermore, homocysteine can also be catabolised via the transsulfuration pathway, leading to production of cysteine and its derivatives, GSH, taurine and inorganic sulfur. The carbon skeleton of methionine, α-ketobutyrate, is eventually oxidatively decarboxylated and enters the TCA cycle to be used for gluconeogenesis.

There may be species differences in methylation metabolism, as SAH was found at higher concentrations in dogs compared with cats, indicating uncompensated flux through the S-adenosylmethionine methylation pathway in dogs. Methylthioadenosine (MTA) was not different between species at baseline and did not change for dogs after free-choice feeding for 28 days, perhaps indicating that the methionine (increased methionine ratio in dogs compared with cats; discussed under amino acids) was being routed to methylation rather than polyamine production. It may be that more limited concentrations of methylated glycines in dogs compared with cats contributes to increased SAH concentrations in dogs, although subsequent studies will be required to confirm this.

### Gastrointestinal microbial metabolites

Concentrations of 38 circulating gastrointestinal microbial metabolites were also evaluated over the 28-day period when dogs and cats were allowed to consume their preferred macronutrient intake (Table 5). In dogs, no microbial metabolite concentrations in blood changed significantly after consuming their preferred macronutrient intake for 28 days. In cats, six circulating microbial metabolite concentrations significantly increased after consuming their preferred macronutrient intake for 28 days: 3-indoxyl sulfate, 4-ethylphenyl sulfate, 2-ethylphenyl sulfate, o-cresol sulfate, 1,2,3-benzenetriol sulfate (2) and equol sulfate. In cats, 10 microbial metabolite concentrations significantly decreased after consuming their preferred macronutrient intake for 28 days: 2-aminophenol sulfate, 3-(4-hydroxyphenyl)propionate, 3-hydroxy-3-phenylpropionate, 4-acetylphenol sulfate, 4-hydroxyphenyl pyruvate, hydroquinone sulfate, methyl-4-hydroxybenzoate sulfate, 3-(4-hydroxyphenyl)lactate, phenylacetate and phenylacetylcarnitine. Thus, no circulating microbial metabolite concentrations changed over the 28-day free-choice feeding period in dogs, but 16/38 (42%) changed in cats (*P*<0.01).

**Table 5.**
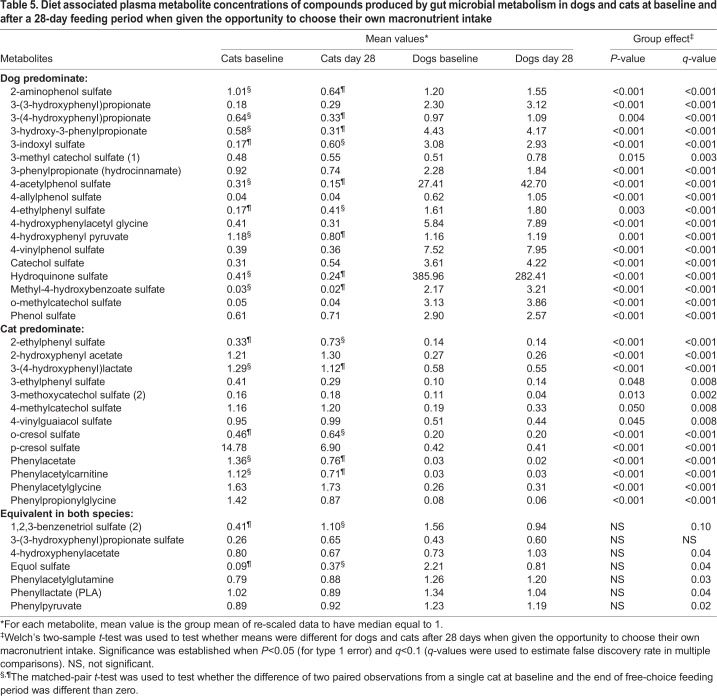
**Diet associated plasma metabolite concentrations of compounds produced by gut microbial metabolism in dogs and cats at baseline and after a 28-day feeding period when given the opportunity to choose their own macronutrient intake**

After consuming their preferred macronutrient intake for 28 days, dogs exhibited altered concentrations for 31/38 microbial metabolites. Dogs had increased concentrations for 18 microbial metabolites compared with cats: 2-aminophenol sulfate, 3-(3-hydroxyphenyl)propionate, 3-(4-hydroxyphenyl)propionate, 3-hydroxy-3-phenylpropionate, 3-indoxyl sulfate, 3-methyl catechol sulfate (1), 3-phenylpropionate (hydrocinnamate), 4-acetylphenol sulfate, 4-allylphenol sulfate, 4-ethylphenyl sulfate, 4-hydroxyphenylacetyl glycine, 4-hydroxyphenyl pyruvate, 4-vinylphenol sulfate, catechol sulfate, hydroquinone sulfate, methyl-4-hydroxybenzoate sulfate, o-methylcatechol sulfate and phenol sulfate. After consuming their preferred macronutrient intake for 28 days, dogs exhibited decreased concentrations for 13 microbial metabolites compared with cats: 2-ethylphenyl sulfate, 2-hydroxyphenyl acetate, 3-(4-hydroxyphenyl)lactate, 3-ethylphenyl sulfate, 3-methoxycatechol sulfate (2), 4-methylcatechol sulfate, 4-vinylguaiacol sulfate, o-cresol sulfate, p-cresol sulfate, phenylacetate, phenylacetylcarnitine, phenylacetylglycine and phenylpropionylglycine.

In summary, concentrations of many sulfated and other microbial catabolic products (31 of 38) were significantly different between dogs and cats, which likely reflects a composite of the species' specific microbiome functional capacity, the pets' metabolic predilections and the diet selections made during the course of this study. Intriguingly, in the cases where dogs had higher concentrations of microbial metabolites compared with cats, the magnitude of those increases were much greater in dogs than the magnitude of increases for those metabolites that were of higher concentration in cats (e.g. 4-acetylphenol sulfate, 4-vinylphenol sulfate and hydroquinone sulfate).

It is well known that modification of the diet affects the colonic microbiota. However, after the 28-day free-choice feeding period, none of the circulating plasma microbial metabolites under consideration changed in dogs, whereas 16 metabolite concentrations changed in cats.

The colonic bacteria produce many compounds that are absorbed and normally excreted in the urine (Tanaka et al., 2015). Evidence suggests that some of the colon-derived uremic solutes are toxic (Tanaka et al., 2015). For example, p-cresol sulfate is derived from p-cresol, an end product of protein catabolism, which is produced in the intestine from tyrosine and phenylalanine by intestinal bacteria (Vanholder et al., 1999). p-Cresol is sulfated to produce p-cresol sulfate in the intestinal wall (Martinez et al., 2005; Schepers et al., 2007; Vanholder et al., 2011). In humans, high concentrations of p-cresol sulfate have been correlated with cardiovascular diseases and mortality in patients with chronic kidney disease (Bammens et al., 2006; Liabeuf et al., 2010; Wu et al., 2012). The renal toxicity of p-cresol sulfate results from its intracellular accumulation, leading to the production of reactive oxygen species, which then trigger induction of inflammatory cytokines that are involved in renal fibrosis (Watanabe et al., 2013). In our study p-cresol sulfate was higher in cats compared with dogs, but did not change in cats as a result of the 28-day free-choice feeding period.

Production of the anti-inflammatory microbial metabolite equol typifies the complex relationship between host species and their microbiomes. Deglycosylation of the dietary flavonoid-glycoside daidzin by hindgut bacteria produces daidzein aglycone, which is subject to subsequent metabolic transformations by both host and microbiome (Rafii, 2015). Daidzein may be absorbed from the gastrointestinal tract and sulfated by the host to daidzein sulfate before circulation and excretion. Alternatively, daidzein may be further metabolized by colonic microbes to equol, which is then absorbed and sulfated to produce equol sulfate. Both daidzein sulfate and equol sulfate were detected in the metabolomics screen reported here. Whereas plasma concentrations of daidzein sulfate were higher in cats at day 28 (least squared means: feline, 0.38; canine, 0.09; *P*<0.01, *q*<0.002), plasma equol sulfate concentrations did not differ between species (Table 5), indicating that although the cats' pattern of food intake might have provided more substrate for microbial production of equol, that potential for production wasn't realized by the feline microbiome. More studies are needed to investigate the health effects of increasing or decreasing the concentrations of other microbial catabolic products (Rehman, 2012).

## Conclusions

The objective of this study was to determine differences between circulating metabolites of dogs and cats after they were given the opportunity to choose their own macronutrient intake for 28 days. We showed that food choices have an effect on subsequent circulating metabolite concentrations. Regarding amino acids, cats had higher concentrations of the essential amino acids histidine, isoleucine, phenylalanine and valine, but lower concentrations of lysine, methionine and threonine compared with dogs. Overall, non-essential amino acids were higher in concentration in cats compared with dogs, and they tended toward glucogenic rather than ketogenic metabolic potential. Regarding lipids, the food choices that cats made increased concentrations of the highly PUFA, AA, EPA and DHA, although AA concentrations were consistently higher in dogs compared with cats. There were intriguing species differences in the balance of complex lipid head groups, such that choline, inositol and ethanolamine moieties were differentially represented in dogs compared with cats. Regarding carbohydrates, cats had increased lactate and decreased pyruvate concentrations after consuming their food intake of choice for 28 days, whereas in dogs concentrations did not change. Glucose homeostasis was maintained regardless of preferred macronutrient intake, although cats may leverage the Cori cycle for lactate utilization to a greater extent than dogs. Cats had higher concentrations of urea compared with dogs, although urea concentrations decreased after 28 days of free-choice food intake. Regarding antioxidants, after the 28-day free-choice feeding period, dogs had marginally higher plasma α-tocopherol concentrations relative to cats (∼20%), yet an exacerbated increase in oxidized tocopherol byproducts; this trend was present at baseline as well, and mitigated by dietary selections. For the endogenous tripeptide-antioxidant glutathione, cats had higher plasma concentrations of both oxidized and reduced forms, as well as higher concentrations of opthalmate. The latter finding suggests a perceived metabolic requirement for higher concentrations of glutathione in cats that can't be met with available cysteine. In contrast, dogs had higher concentrations of metabolites that indicated increased transsulfuration flux (AHB) and increased production of metabolites that might decrease export and recycling of glutathione into the plasma (5-oxoproline, oxidized cys-gly). Together these findings suggest species differences in the metabolic regulation of an endogenous, protein-derived antioxidant when pets self-regulate their protein intake. Cats also had consistently higher concentrations of components of methylation metabolism. Regarding circulating microbial metabolites, in general there was more stability over the 28-day free-choice feeding period for dogs compared with cats. No circulating microbial metabolite concentrations changed significantly over 28 days in dogs, however 16/38 changed in cats, with some metabolites concentrations increasing and some decreasing. In conclusion, metabolomic analysis revealed metabolite differences between dogs and cats, and these differences reflected the differences in food choices between species as well as metabolic tendencies.

## MATERIALS AND METHODS

### Animals and ethics statement

All study protocols and this study were reviewed and approved by the Institutional Animal Care and Use Committee, Hill's Pet Nutrition, Inc., Topeka, KS, USA (Permit Numbers: canine 590 and feline 577), and complied with the National Institutes of Health guide for the care and use of laboratory animals (NIH Publications No. 8023, revised 1978). All studies were conducted at the Hill's Pet Nutrition Center.

### Foods

Prior to beginning the study, all dogs and cats had been fed many types of commercial and non-commercial foods of varying nutrient compositions, including dry and canned foods, in palatability studies. All foods met the requirements established by AAFCO for complete and balanced pet foods for adult dogs and cats. Although foods with more extreme carbohydrate, protein and fat concentration are available in the marketplace, the macronutrient content of the offered foods reflected the variation of pet foods available currently.

To determine that foods were of similar palatability, different groups of dogs and cats than those used in this study were utilized for palatability equivalency testing as previously reported (Hall et al., 2018). Ultimately, palatability was masked both by changing macronutrient sources as well as concentrations of palatability enhancers (Hall et al., 2018).

Dog foods for this study were produced by Hill's Pet Nutrition, Inc., and met the nutritional requirements for adult dogs (≥1 year). Food was available in dry form only. Macronutrient composition of foods was determined by a commercial laboratory (Eurofins Scientific, Inc., Des Moines, IA, USA). Proximate analyses were completed using the following techniques: moisture-AOAC 930.15; protein-AOAC 2001.11; fat-AOAC 954.02; fiber-AOAC 962.09; and ash-AOAC 942.05. Carbohydrate composition was determined by calculation. Macronutrient composition, expressed as percentage of food, as fed, is shown in Table 6.

**Table 6.**
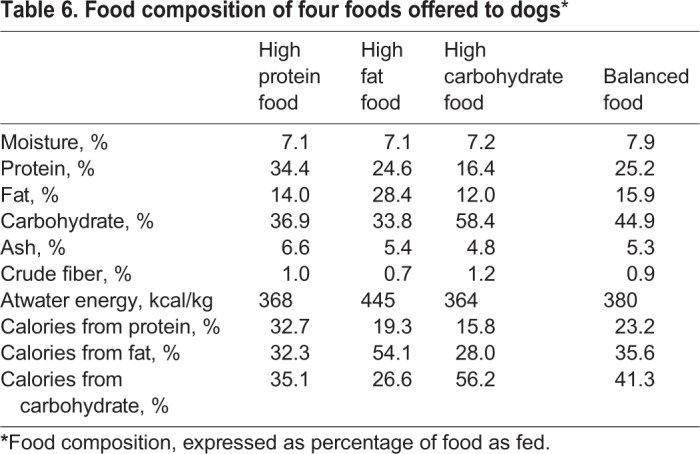
Food composition of four foods offered to dogs*

Cat foods were produced by Hill's Pet Nutrition, Inc., and met the nutritional requirements for adult cats (≥1 year). Food was available in dry form only. Macronutrient composition of foods was determined by a commercial laboratory (Eurofins Scientific, Inc.). Carbohydrate composition was determined by calculation. Macronutrient composition, expressed as percentage of food, as fed, is shown in Table 7. No ingredient sources of EPA or DHA were added to the food.

**Table 7.**
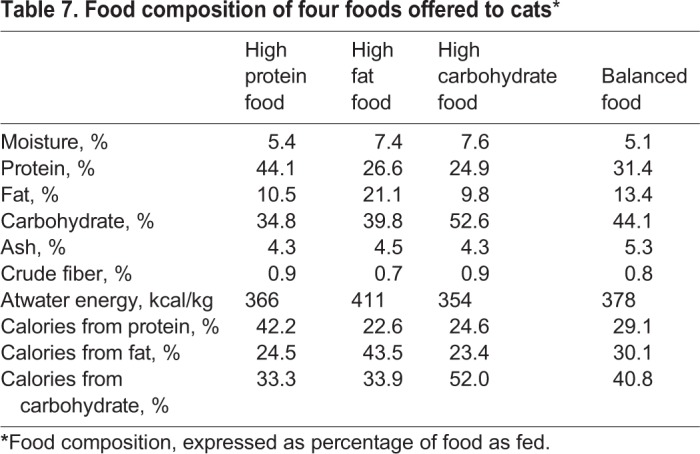
Food composition of four foods offered to cats*

For dog and cat foods, changes to the macronutrient profile necessitated changes in ingredient composition. However, with the exception of macronutrients, the nutrient profiles of all foods were balanced such that similar amounts of vitamins and minerals were consumed without regard to macronutrient changes.

### Dogs

These studies were conducted using 17 dogs randomly selected from a colony of Beagles (inclusion criteria were availability; exclusion criteria were preexisting illness and previously documented food bowl position bias); ranging in age from 2.1 to 11.8 years [mean±s.d. (standard deviation), 7.4±3.1 years]; 12 ovariohysterectomized females and five neutered males; and with varying lean body and fat body masses (body weight, mean±s.d., 10.9±1.50 kg, range, 7.6-13.6 kg; body lean, 6.70±1.16 kg, range, 4.88-9.33 kg; body fat, 3.78±0.94 kg, range, 2.4-6.2 kg). All dogs were exercised daily and were provided with regular opportunities for socialization and environmental enrichment. Dogs were paired and housed in runs for 15 h each day, with space for sleeping and exercise. During the day they were housed together with 10 and 7 dogs per room, with 45 min intervals alternating between the group rooms and outside play in the dog park.

Dogs were fed once daily and were moved to individual stalls for a 1 h feeding period. Dogs were allowed to choose freely among any of the four foods, which were offered in a line at the same time, but were limited in food intake to a predetermined caloric allowance by restricting food intake once allowed caloric consumption was attained. Similar to the palatability studies defined above, the canine four-bowl test reported here had a bowl for each of the four foods available until the dogs had consumed the allotted daily calories. Multiple bowl testing is a common practice in the pet food industry to evaluate food intake choices. Each dog's caloric consumption was based on age, body weight and activity level. Dogs were microchipped and food disappearance was recorded from all food bowls, which were set on scales. A computer calculated change in food weight and thus calories consumed. Thus, the total amount of food consumed was controlled to meet daily metabolic energy requirements and maintain body weight. The amount of each food consumed from each food source was used to calculate daily and average 28-day composite macronutrient intake for each dog.

### Cats

Studies were conducted using 27 cats randomly selected from a colony of cats (inclusion criteria were availability; exclusion criteria were pre-existing illness); ranging in age from 2.3 to 6.6 years (mean±s.d., 4.0±1.6 years); 14 ovariohysterectomized females and 13 neutered males; and with varying lean body and fat body masses (body weight, mean±s.d., 5.42±0.93 kg, range, 3.6-7.1 kg; body lean, 3.92±0.54 kg, range, 2.9-4.8 kg; body fat, 1.36±0.46 kg, range, 0.3-2.2 kg). Cats were housed in indoor rooms of 10 and 17, with access to glass-enclosed porches and toys at all times.

Cats were housed together, with 13 to 14 cats per room. There were four feeding stations in each room. From the beginning of the study, cats were offered food *ad libitum* from any of the four feeding stations in the room. Multiple bowl testing is a common practice in the pet food industry to evaluate food intake choices. Similar to dogs, cats were micro-chipped such that food disappearance was recorded from each feeding station after every meal. Cats were allowed to choose freely among any of the four foods offered, but were limited in food intake to a predetermined caloric allowance based on age and body weight. Thus, the total amount of food consumed was controlled to meet daily metabolic energy requirements and maintain body weight. The amount of each food consumed from each food source was used to calculate daily and average 28-day composite macronutrient intake for each cat.

### Study design and measurements

For a 28-day period, dogs and cats were given the opportunity to choose from any of four completely balanced foods that differed in concentration of macronutrients as a percent of total calories fed. Dogs and cats were allowed to choose among the four food sources until the amount of food consumed met the daily established caloric allowance to maintain a healthy weight. Based on the amount of food consumed from each feeding station, the total amount of protein, fat and carbohydrate consumed each day were calculated and summed over the 28-day period. The percent of each macronutrient consumed relative to total caloric intake over the same 28 day period was then determined.

All foods had been previously balanced for palatability. Animals that exhibited a bowl position bias in previous studies were excluded from this study. Furthermore, bowl position was changed daily such that animals needed to move their consumption position in order to maintain the macronutrient intake mixture of choice. Their macronutrient intake mixture stabilized within the first week and was stable for the remainder of the study.

Blood was collected at 28 days after animals had consumed their food intake composition of choice in order to determine plasma metabolomic profiles. Blood was collected from dogs before the next day's meal resulting in a 23 to 24 h food withholding period before the blood sample was drawn. For blood collection in cats, food bowls were removed at the end of the day and blood was collected before food was replaced the next day such that food was withheld 15 to 16 h before blood was collected. Plasma metabolites were measured by a commercial laboratory (Metabolon, Durham, NC, USA). Extracted supernatant was split and run on gas chromatography and liquid chromatography mass spectrometer platforms (Evans et al., 2009) in a randomized order. Gas chromatography (for hydrophobic molecules) and liquid chromatography (for hydrophilic molecules) were used to identify and provide relative quantification of small metabolites present in plasma samples. Endogenous biochemicals included amino acids, peptides, carbohydrates, lipids, nucleotides, cofactors and vitamins. Data for each individual compound were normalized by calculating the median values for each run-day block (block normalization). This minimized any inter-day instrument gain drift, but did not interfere with intra-day sample variability. Missing values were assumed to be below the level of detection for that compound with the instrumentation used. Missing values (if any) were imputed with the observed minimum for that particular compound. Imputed values were added after block-normalization. The complete dataset for canine and feline plasma metabolites is shown as a heat map of statistically significant biochemicals profiled in this study (Table S1).

### Statistical analyses

Analyses were performed using ArrayStudio (Omicsoft Corporation, Cary, NC, USA) on log transformed data. The matched-pair *t*-test was used to test whether the difference of two paired observations from a single pet at baseline and the end of free-choice feeding period was different than zero. Welch's two-sample *t*-test was used to test whether means were different for dogs and cats after 28 days when given the opportunity to choose their own macronutrient intake. This test allows for unequal variances and has an approximate *t*-distribution with degrees of freedom estimated using Satterthwaite's approximation. Significance was established when *P*<0.05 (for type 1 error) and *q*<0.1 (*q*-values were used to estimate false discovery rate in multiple comparisons). The Chi-square test was used to compare overall change in gastrointestinal microbial metabolite concentrations in dogs and cats after 28 days of free-choice food intake.

## Supplementary Material

Supplementary information
