# Supplementary material for: Comparison of circulating metabolite concentrations in dogs and cats when allowed to freely choose macronutrient intake
Source: Biol Open. 2018 Sep 26;7(11):bio036228. doi: 10.1242/bio.036228 (PMC6262854; doi:10.1242/bio.036228)
Supplement: Supplementary information [file biolopen-7-036228-s1.pdf]

**Table S1.** Heat map of the complete dataset for canine and feline plasma metabolites. Red and green shaded cells indicate P = 0.05 (red indicates that the mean values are significantly higher for that comparison; green values are significantly lower). Light red and light green shaded cells indicate 0.05 < P < 0.10 (light red indicates that the mean values trend higher for that comparison; light green values trend lower).

Click here to Download Table S1
